# Supplementary material for: Multi-metric evaluations of acute psychedelic effects on fMRI brain entropy
Source: Nat Commun. 2026 Jun 24;17:7940. doi: 10.1038/s41467-026-74215-5 (PMC13448839; doi:10.1038/s41467-026-74215-5)
Supplement: Supplementary file 1 [file 41467_2026_74215_MOESM12_ESM.pdf]

nature portfolio

Corresponding author(s): Patrick MacDonald Fisher

Last updated by author(s): Jul 17, 2023

Reporting Summary

Nature Portfolio wishes to improve the reproducibility of the work that we publish. This form provides structure for consistency and transparency in reporting. For further information on Nature Portfolio policies, see our Editorial Policies and the Editorial Policy Checklist.

Statistics

For all statistical analyses, confirm that the following items are present in the figure legend, table legend, main text, or Methods section.

n/a	Confirmed	
☐	☒	The exact sample size (*n*) for each experimental group/condition, given as a discrete number and unit of measurement
☐	☒	A statement on whether measurements were taken from distinct samples or whether the same sample was measured repeatedly
☐	☒	The statistical test(s) used AND whether they are one- or two-sided<br>*Only common tests should be described solely by name; describe more complex techniques in the Methods section.*
☐	☒	A description of all covariates tested
☐	☒	A description of any assumptions or corrections, such as tests of normality and adjustment for multiple comparisons
☐	☒	A full description of the statistical parameters including central tendency (e.g. means) or other basic estimates (e.g. regression coefficient) AND variation (e.g. standard deviation) or associated estimates of uncertainty (e.g. confidence intervals)
☐	☒	For null hypothesis testing, the test statistic (e.g. *F*, *t*, *r*) with confidence intervals, effect sizes, degrees of freedom and *P* value noted<br>*Give P values as exact values whenever suitable.*
☒	☐	For Bayesian analysis, information on the choice of priors and Markov chain Monte Carlo settings
☒	☐	For hierarchical and complex designs, identification of the appropriate level for tests and full reporting of outcomes
☐	☒	Estimates of effect sizes (e.g. Cohen's *d*, Pearson's *r*), indicating how they were calculated

Our web collection on statistics for biologists contains articles on many of the points above.

Software and code

Policy information about availability of computer code

Data collection	no software used
Data analysis	BOLD data were preprocessed in SPM12 (http://www.fil.ion.ucl.ac.uk/spm) and denoised using the CONN toolbox using MATLAB (The Mathworks, inc., version 2019b 9.7.0). Subsequent analysis scripts are available at https://github.com/anders-s-olsen/CopBET

For manuscripts utilizing custom algorithms or software that are central to the research but not yet described in published literature, software must be made available to editors and reviewers. We strongly encourage code deposition in a community repository (e.g. GitHub). See the Nature Portfolio guidelines for submitting code & software for further information.

Data

Policy information about availability of data

All manuscripts must include a data availability statement. This statement should provide the following information, where applicable:

- Accession codes, unique identifiers, or web links for publicly available datasets
- A description of any restrictions on data availability
- For clinical datasets or third party data, please ensure that the statement adheres to our policy

We shared relevant analysis scripts with original authors, hoping to ensure as much as possible that our computations aligned with original reports; we are thankful for the feedback we received. All functions used to derive entropy estimates from preprocessed data have been compiled into the "Copenhagen Brain Entropy Toolbox" (CopBET), a Matlab-based toolbox that can be found here: https://github.com/anders-s-olsen/CopBET. The permutation testing code is also available here. Code for other statistical analyses and figures can be made available upon request. Data is stored in the cimbi database and may be made available upon reasonable

request.

Research involving human participants, their data, or biological material

Policy information about studies with human participants or human data. See also policy information about sex, gender (identity/presentation), and sexual orientation and race, ethnicity and racism.

Reporting on sex and gender	Findings apply to both sexes, the sample contained 18 males and 10 females. Linear models were adjusted with sex as a covariate. We do not have permission to share individual-level data. Sex and gender based analyses were not considered as there is no indication that psychedelic effects on brain entropy are variable between sexes or genders and this sample was underpowered to evaluate such a distinction.
Reporting on race, ethnicity, or other socially relevant groupings	Race and ethnicity were not evaluated within this sample.
Population characteristics	Age was considered as a relevant covariate and was thus included in linear models. All participants were physically and mentally healthy. Age Mean (SD) Range = 32.5 (8) 24-58.
Recruitment	Participants were recruited from a database of individuals interested in participating in a study involving psychedelics. They were also screened for the presence of somatic and mental illness and mental illness in a first degree relative. Thus, they constitute a particularly healthy population. Furthermore, there is a potential selection bias for individuals who are more adventurous as they volunteered to take part in psychedelic research.
Ethics oversight	The ethics committee of the capital region of Copenhagen and the Danish Medicines Agency

Note that full information on the approval of the study protocol must also be provided in the manuscript.

Field-specific reporting

Please select the one below that is the best fit for your research. If you are not sure, read the appropriate sections before making your selection.

☒ Life sciences ☐ Behavioural & social sciences ☐ Ecological, evolutionary & environmental sciences

For a reference copy of the document with all sections, see nature.com/documents/nr-reporting-summary-flat.pdf

Life sciences study design

All studies must disclose on these points even when the disclosure is negative.

Sample size	Sample size (28) was determined due to previous publications evaluating the acute effects of psilocybin on fMRI outcomes.
Data exclusions	Of the 130 resting-state fMRI scan sessions acquired during psilocybin intervention days for the 28 participants (76 Scanner A and 54 Scanner B), Nine scans and no participants were excluded from analyses: two scans from one participant because of excessive head motion through the scan session (>50% of volumes flagged for censoring), one scan from one participant due to technical problems with data acquisition, two scans from one participant due to 1) interrupted scan and 2) technical problems with data acquisition, one scan each from three participants due to interrupted scans. Thus, 121 scan sessions were included in analyses. One scan session did not have an accompanying measure of PPL, another was without a measure of SDI, these individual scans were excluded from these respective analyses. Such exclusions are in line with the preregistration as described here https://aspredicted.org/bw8y7.pdf
Replication	This manuscript evaluates the replicability of other entopy metrics as biomarkers of psychedelic drug effects. The association between fMRI entropy metrics and plasma drug levels, subjective drug intensity and serotonin 2A receptor occupancy (estimated from plasma psilocin level) were all estimated and only findings that were consistent across all three were considered statistically significant.
Randomization	This study was a single-blinded crossover study. Subjects were randomly allocated to receive either psilocybin or ketanserin first. Participants were balanced roughly by age and sex.
Blinding	Participants were blinded to the drug they received.

Reporting for specific materials, systems and methods

We require information from authors about some types of materials, experimental systems and methods used in many studies. Here, indicate whether each material, system or method listed is relevant to your study. If you are not sure if a list item applies to your research, read the appropriate section before selecting a response.

Materials & experimental systems

n/a	Involved in the study
[x]	[ ] Antibodies
[x]	[ ] Eukaryotic cell lines
[x]	[ ] Palaeontology and archaeology
[x]	[ ] Animals and other organisms
[ ]	[x] Clinical data
[x]	[ ] Dual use research of concern
[x]	[ ] Plants

Methods

n/a	Involved in the study
[x]	[ ] ChIP-seq
[x]	[ ] Flow cytometry
[ ]	[x] MRI-based neuroimaging

Clinical data

Policy information about clinical studies

All manuscripts should comply with the ICMJE guidelines for publication of clinical research and a completed CONSORT checklist must be included with all submissions.

Clinical trial registration: NCT03289949

Study protocol: Study protocol can be found here https://nru.dk/index.php/allcategories/category/83-h-16026898-den-neurobiologiske-effekt-af-5-ht2a-receptor-modulering-np2

Data collection: Data collection was performed at University Hospital Copenhagen, Rigshospitalet between 2018 and 2021

Outcomes: Primary outcome for this data was described as "Effects of psilocybin and ketanserin on brain function assessed with fMRI and PET". This was assessed through the lens of fMRI "brain entropy".

Magnetic resonance imaging

Experimental design

Design type: task-free with and without drug

Design specifications: each participant was scanned once before drug administration and several times after (range = 2-7)

Behavioral performance measures: Participants reported their subjective drug intensity orally after each scan on a scale from 0 (no effects) to 10 (very intense)

Acquisition

Imaging type(s): functional

Field strength: 3

Sequence & imaging parameters: Due to logistical constraints, MRI scans were acquired on one of two Siemens 3T MAGNETOM Prisma scanners (“Scanner A”, “Scanner B”). The first 15 participants were scanned on Scanner A, the final 13 participants were scanned on Scanner B, all participants completed all scans on a single scanner.

A high-resolution, T1-weighted 3D MPRAGE structural image was acquired (Scanner A: inversion time = 900 ms, TE = 2.58 ms, TR = 1900 ms, flip angle = 9°, in-plane matrix = 256x256, resolution = 0.9x0.9 mm, 224 slices; slice thickness = 0.9 mm, no gap, Scanner B: inversion time = 920 ms, TE = 2.41 ms, TR = 1810 ms, flip angle = 9°, in-plane matrix = 288 × 288, in-plane resolution = 0.8×0.8 mm, number of slices = 224, slice thickness = 0.8 mm).

BOLD fMRI data was obtained using a T2*-weighted gradient echo-planar imaging (EPI) sequence.

Scanner A: 64-channel head coil, TR = 2000 ms, TE = 30 ms, flip angle = 90°, in-plane matrix = 64x64 voxels, in-plane resolution=3.6x3.6 mm, number of slices = 32, slice thickness = 3.0 mm, gap = 0.75 mm, phase-encoding direction = AP, scan time = 10 minutes, volumes = 300.

Scanner B: 32-channel head coil, TR = 800 ms, TE = 37 ms, flip angle = 52°, in-plane matrix = 104x104 voxels, in-plane resolution = 2x2 mm, slices = 72, slice thickness = 2.0 mm, gap = 0, multi-band acceleration factor = 8, scan time = 10 minutes, volumes = 750. Due to an acquisition error, 8 participants from Scanner B were scanned for only five minutes (i.e., 375 volumes).

Area of acquisition: whole-brain

Diffusion MRI: [ ] Used [x] Not used

Preprocessing

Preprocessing software	BOLD data were preprocessed in SPM12 (http://www.fil.ion.ucl.ac.uk/spm) and denoised using the CONN toolbox 2 using MATLAB (The Mathworks, inc., version 2019b 9.7.0). Preprocessing pipelines were identical for both scanners unless specified. We applied slice-timing correction (scanner A only because scanner B used multi-band), data were unwarped and realigned, structural scans were co-registered to functional data. High-resolution structural images were segmented into grey-matter, white-matter and CSF-maps; functional data were normalised to MNI152 space based on parameters estimated during the segmentation procedure and smoothed using a 6mm FWHM gaussian kernel.
Normalization	functional data were normalised to MNI152 space based on parameters estimated during the segmentation procedure
Normalization template	MNI152
Noise and artifact removal	Denoising included aCompCor regression of white-matter and CSF time-series (first five components for each and their first derivatives) 3 as well as regression of 6-motion parameters and their first derivatives, and scrubbing of volumes flagged using the artifact detection tool (ART, https://www.nitrc.org/projects/artifact_detect). Some analyses utilised unsmoothed data to align with previous methods (Intra-network synchrony, Motif-connectivity distribution, Multi-scale Sample Entropy.). A bandpass filter of 0.008 - 0.09 Hz was applied. Regional time-series were averaged in CONN using analysis-specific atlases. Following preprocessing and denoising and to align the temporal frequency between the two scanners, scanner B time-series were temporally downsampled to match the TR of scanner A (i.e., 2s) using the MATLAB command "downsample". To explore the effect of denoising decisions on the associations between PsiFx and brain entropy metrics, analyses were repeated for six additional pre-processing pipelines. Each pre-processing pipeline was run on the data parcellated as described in the section "Effect of parcellation" i.e., 116 ROIs assigned to seven networks. Each pipeline changed one variable from the main pipeline. These were as follows: 1) adding global signal regression, 2) removing the low-pass 0.09 Hz filter (i.e., not removing high-frequency signal), 3) expanding the 12-motion regressors to include squares of the derivatives (i.e., Volterra expansion), 4) not regressing out flagged volumes 5) regressing flagged volumes with a stricter threshold (z >3 or motion>0.5mm), (6) applying a narrower bandpass filter (0.03-0.07 Hz).
Volume censoring	In standard pipeline: Volumes with framewise global signal deviations greater than 4 SDs or displacement greater than 2 mm using the ART toolbox

Statistical modeling & inference

Model type and settings	Brain entropy quantifications based on static connectivity matrixes, dynamic connectivity estimates and dynamic activity timecourses
Effect(s) tested	Association between brain entropy metrics and drug effects were evaluated using mixed linear effects models

Specify type of analysis: ☐ Whole brain ☐ ROI-based ☒ Both

Anatomical location(s): Analysis frameworks were all based on previous publications

Statistic type for inference (See Eklund et al. 2016)	ROI-or network-wise
Correction	Max-T correction

Models & analysis

n/a	Involved in the study
☐	☒ Functional and/or effective connectivity
☐	☒ Graph analysis
☒	☐ Multivariate modeling or predictive analysis

Functional and/or effective connectivity	Functional connectivity (Pearson's correlations and partial correlations)
Graph analysis	Both weighted and binarised subject level graphs were used. Entropy of out-network connectivity, degree distribution and path-length distribution were evaluated.
