# Supplementary material for: Multi-metric evaluations of acute psychedelic effects on fMRI brain entropy
Source: Nat Commun. 2026 Jun 24;17:7940. doi: 10.1038/s41467-026-74215-5 (PMC13448839; doi:10.1038/s41467-026-74215-5)
Supplement: Supplementary file 2 [file 41467_2026_74215_MOESM13_ESM.pdf]

nature portfolio

Peer Review File

Multi-metric evaluations of acute psychedelic effects on fMRI brain entropy

Corresponding Author: Dr Patrick Fisher

This manuscript has been previously reviewed at another journal. This document only contains information relating to versions considered at Nature Communications.

This file contains all reviewer reports in order by version, followed by all author rebuttals in order by version.

Version 1:

Reviewer comments:

Reviewer #1

(Remarks to the Author)
The manuscript addresses a critical and timely question in psychedelic neuroscience: whether psilocybin reliably alters brain entropy as measured by fMRI, and which metrics best capture these effects. Building on the Entropic Brain Hypothesis (EBH), the authors systematically assess various previously reported entropy measures in an independent cohort with repeated scans and pharmacokinetic as well as subjective correlates. These metrics span static functional connectivity (correlation matrices), dynamic connectivity (time-varying relationships between time series), and dynamic activity (entropy of regional time series), offering a comprehensive evaluation beyond single-metric, single-scan designs. The inclusion of multiple preprocessing pipelines, scanner effects, and inter-metric correlations adds rigor and transparency, positioning this work as a benchmark for future studies. The dataset is both useful and timely, and the article—ambitious and interesting—represents a significant improvement since its earlier version.

The manuscript is methodologically exhaustive—bordering on too comprehensive at times—but this rigor ultimately strengthens the work. Importantly, the authors have wisely placed a substantial portion of the detailed results in the supplementary materials, which preserves readability and flow in the main text. I fully approve the inclusion of these additional analyses, as they enhance robustness and transparency.

My only recommendation concerns the results and discussion sections writing style: the current narrative occasionally feels syncopated, with abrupt transitions that could benefit from smoother linking and clearer signposting (more detail below).

The introduction is well-grounded in prior literature, clearly articulating the clinical and theoretical relevance of psychedelics and entropy-based models. EBH is explained concisely, and the mathematical basis of entropy (Shannon entropy and alternatives) is appropriately introduced. The rationale for grouping metrics into static connectivity, dynamic connectivity, and dynamic activity is logical and aids conceptual clarity. The theoretical context could be expanded to include alternative models (e.g., REBUS, network-control theory) earlier, setting up the discussion for later integration.

The results section is impressively detailed and transparent, reporting both significant and null findings across all metrics. Robustness checks (scanner, parcellation, denoising pipelines) and inter-metric correlations are valuable additions that strengthen interpretability. However, the narrative lacks conceptual synthesis: readers must piece together which metrics matter most and why. A short summary paragraph at the start or end of the Results would help.

The discussion section has a comprehensive coverage of replication successes, novel findings, null results, methodological limitations, and future directions. The acknowledgment of limitations is transparent and future recommendations for multimodal validation, standardized pipelines, are all appropriate. The biggest problem of the manuscript in my opinion is the fragmentation of the discussion section which reads like an extended results commentary rather than a conceptual synthesis.

Theoretical integration is underdeveloped: EBH is mentioned but not critically evaluated in light of findings, nor are alternative models explicitly linked to results. Null findings are acknowledged but not deeply interpreted—do they challenge

EBH? The theoretical context could be expanded to include alternative models (e.g., REBUS, network-control theory).

(Remarks on code availability)

Reviewer #2

(Remarks to the Author)
The authors have addressed all of my comments.

(Remarks on code availability)

Reviewer #4

(Remarks to the Author)
This paper provides a comprehensive comparison of the psychedelic effects on various measures of brain entropy derived from fMRI data. Psychedelic effects on brain activity have been a topic of growing interest, with increasing evidence suggesting potential clinical benefits in treating psychiatric disorders. Entropy is a useful metric for characterizing dynamic changes in brain states and their temporal evolution. This study recruited a new cohort to serve as an independent test sample for examining brain entropy under the influence of a psychedelic drug, which is an important strength. The authors further assessed the impact of fMRI preprocessing choices and compared 14 different entropy metrics—an analysis that has not been previously conducted. A strength is the use of longitudinal design. The comprehensive comparison is considered a strength but raises a concern of multiple comparison correction though many of metrics are quite different in nature. Below are the major and minor concerns.
Novelty is considered moderate though confirmation studies are also important but would ideally be based on more controlled data with a good sample size.
Although these metrics are all labeled as entropy measures, many differ substantially in their underlying definitions. For example, some capture temporal entropy, whereas others characterize entropy of spatial relationships between brain regions or the topological properties of connectomes. Some are related to Shannon entropy, while others reflect differential entropy. Therefore, obtaining different or inconsistent results across these metrics is not surprising.
Data acquired from Scanner B has much higher spatial and temporal resolution and longer time series than those acquired from Scanner A. Entropy measures are highly sensitive to these parameters when applied to fMRI data. For example, the histogram-based Shannon entropy, LZ, and Sample En will have a systematic difference if the time series length is different. Higher temporal resolution will capture higher frequency signal. As entropy reflects irregularity, higher temporal resolution likely will provide more stable entropy estimates. This contrast can be seen from the rsfmri entropy studies based on the traditional non-multi-band bold fmri data and multi-band fmri data such as HCP. I would suggest the authors analyze these two subcohorts separately. Otherwise, many entropy metrics are not combinable given the substantial differences of the data properties. If you want to do that, the only way will be downsampling cohort B to match the data from cohort A though you will still see systematic effects caused by the hemodynamic response function sampling difference, SNR and others. Coils are also very different in terms of SNR and regional inhomogeneity, which can be partially controlled through harmonization for those temporal brain entropy measures.
Many subjects scanned in Scanner B had significantly shorter time series than the others. This will cause big variations to the entropy measures.
There is a positive correlation between the drug intensity and drug level and motions. Likely, part of the observed fmri entropy effects was contributed by the drug-induced data variations. This is in addition to the potential vascular contributions commented by previous reviewers.
Each entropy metric involves empirical parameters. Should the author describe how those parameters were chosen? For connectome binarization, how was the threshold was chosen? LZ depends on a threshold too.
As psychedelic drug impact the whole brain dramatically, the global signal will be affected. Then GSR is really problematic. GSR itself can be an index for assessing the drug effects.
For illustrations, could the authors show the statistical comparison results between pre- and post-drug entropy (at the peak drug level)?
If the difference between NGSC and Von Neumann entropy is the data dimension reduction, why not merge them and use one metric to avoid confusion?
One interesting though might be underpowered question that the authors can explore is to see the value of these metrics for predicting the psychedelic effects through an ablation study.
The last sentence in abstract is obvious based on the different definitions of these metrics.
Variations of the time gap between drug and fMRI should be discussed.
Result summary in Abstract is seemly inconsistent with Discussion. More metrics mentioned in Abstract that have significant positive effects while discussion (line 235).
The timing for acquiring the several fMRI datasets should be provided. If both the timing and the intervals are different across the subjects, they should be modeled in the linear mixed model.
Legends for all plots should mention that all timepoints from all subjects were pooled.
It will be also important to show the curve for all subjects instead of showing the scatter points.
Not sure whether EEG or eye tracking or other biological data such as EKG, heart rate, blood pressures were measured. Maybe the authors can add some discussions about those as they may provide more objective information about the psychedelic drug effects.

It may be interesting to check entropy of the non-denoised data (only with motion correction).
Fig. S1 B shows an obvious outlier. It would be interesting to see the results after excluding that subject.

(Remarks on code availability)
code sharing is a strength

Reviewer #5

(Remarks to the Author)
I co-reviewed this manuscript with one of the reviewers who provided the listed reports. This is part of the Nature Communications initiative to facilitate training in peer review and to provide appropriate recognition for Early Career Researchers who co-review manuscripts.

(Remarks on code availability)

Version 2:

Reviewer comments:

Reviewer #4

(Remarks to the Author)
I appreciate the comprehensive response. While I considered novelty moderate, the paper is important regarding the continuous entropy and PPL monitoring and the results would confirm the link between imaging-derived metrics and neuronal (actually should be quasi-neuronal since vascular effects have not been delineated). I have some minor comments for the authors to consider: I thought including the many different entropy metrics with totally different meanings only confuse the readership. If the authors prefer this way, I would describe them more carefully. Importantly, some of them are entropy of brain activity, the others are secondary and irrelevant to brain activity or at least not directly related. Some of them rather reflect the anatomical or topological construct.

Neurovascular contribution might need a bit more discussion. As shown in Del Mauro et al: https://doi.org/10.1002/jmri.28948Digital, resting state fmri derived brain entropy correlates with physiological indices. By controlling those non-neuronal effects, entropy is still related to cognitive function as previously reported in https://doi.org/10.1016/j.neuroimage.2021.117893. Similarly, the authors might check whether further including those indices in the analysis will affect the effects or not.

Motion handling is considered standard and effective. Meanwhile, motion could also be related to the neuronal drug effects.

Preprocessing in Method section didn't say downsampling. Did I miss something there?

fMRI based brain complexity or entropy analysis has gained increasing interest and several of the early papers should be cited:
Yang et al. Neurobiol Aging 2012, 10.1016/j.neurobiolaging.2012.05.004
Wang et al. Plos one 2014, https://doi.org/10.1371/journal.pone.0089948
Smith et al, Brain imaging behav 2014, 10.1007/s11682-013-9276-6

(Remarks on code availability)

Reviewer #5

(Remarks to the Author)
I co-reviewed this manuscript with one of the reviewers who provided the listed reports. This is part of the Nature Communications initiative to facilitate training in peer review and to provide appropriate recognition for Early Career Researchers who co-review manuscripts.

(Remarks on code availability)

Open Access This Peer Review File is licensed under a Creative Commons Attribution 4.0 International License, which permits use, sharing, adaptation, distribution and reproduction in any medium or format, as long as you give appropriate credit to the original author(s) and the source, provide a link to the Creative Commons license, and indicate if changes were made.
In cases where reviewers are anonymous, credit should be given to 'Anonymous Referee' and the source.
The images or other third party material in this Peer Review File are included in the article's Creative Commons license, unless indicated otherwise in a credit line to the material. If material is not included in the article's Creative Commons license and your intended use is not permitted by statutory regulation or exceeds the permitted use, you will need to obtain permission directly from the copyright holder.
To view a copy of this license, visit https://creativecommons.org/licenses/by/4.0/

We would like to thank the reviewers for their time and effort providing feedback on our manuscript. Please find below a point-by-point response to comments with an indication of associated changes to the manuscript.

Reviewer #1 (Remarks to the Author):

The manuscript addresses a critical and timely question in psychedelic neuroscience: whether psilocybin reliably alters brain entropy as measured by fMRI, and which metrics best capture these effects. Building on the Entropic Brain Hypothesis (EBH), the authors systematically assess various previously reported entropy measures in an independent cohort with repeated scans and pharmacokinetic as well as subjective correlates. These metrics span static functional connectivity (correlation matrices), dynamic connectivity (time-varying relationships between time series), and dynamic activity (entropy of regional time series), offering a comprehensive evaluation beyond single-metric, single-scan designs. The inclusion of multiple preprocessing pipelines, scanner effects, and inter-metric correlations adds rigor and transparency, positioning this work as a benchmark for future studies. The dataset is both useful and timely, and the article—ambitious and interesting—represents a significant improvement since its earlier version.

The manuscript is methodologically exhaustive—bordering on too comprehensive at times—but this rigor ultimately strengthens the work. Importantly, the authors have wisely placed a substantial portion of the detailed results in the supplementary materials, which preserves readability and flow in the main text. I fully approve the inclusion of these additional analyses, as they enhance robustness and transparency.

My only recommendation concerns the results and discussion sections writing style: the current narrative occasionally feels syncopated, with abrupt transitions that could benefit from smoother linking and clearer signposting (more detail below). The introduction is well-grounded in prior literature, clearly articulating the clinical and theoretical relevance of psychedelics and entropy-based models. EBH is explained concisely, and the mathematical basis of entropy (Shannon entropy and alternatives) is appropriately introduced. The rationale for grouping metrics into static connectivity, dynamic connectivity, and dynamic activity is logical and aids conceptual clarity. The theoretical context could be expanded to include alternative models (e.g., REBUS, network-control theory) earlier, setting up the discussion for later integration.

We appreciate the comment on including alternative models. We opted to focus our paper on the Entropic Brain Hypothesis as it posited a straightforward, but nonspecific hypothesis i.e., that psychedelics increase brain entropy but without articulating a specific entropy quantification. By contrast, the REBUS model does not explicitly articulate a hypothesis testable using fMRI entropy quantifications. We are hesitant to speculate on potential relations between REBUS-based fMRI quantifications, e.g., top-down directed connectivity, and the entropy metrics presented in our manuscript. However, we agree that such synthesis is important and have therefore included a sentence in the Discussion indicating that future research may wish to

evaluate the relation between these entropy metrics and other models of psychedelic brain action (l.405-406). Thank you for bringing the potential relation to Network Control Theory to our attention. In preparation for resubmission, we found some intriguing studies linking meta-state complexity, network control theory and Lempel-Ziv complexity of the EEG signal and have added a paragraph to the Discussion contextualising these. (l-339-344). We are apprehensive about dwelling on other models too much here because it may over-complicate the manuscript without a clear benefit nor a clear notion of which additional models we ought to and ought not to introduce.

The results section is impressively detailed and transparent, reporting both significant and null findings across all metrics. Robustness checks (scanner, parcellation, denoising pipelines) and inter-metric correlations are valuable additions that strengthen interpretability. However, the narrative lacks conceptual synthesis: readers must piece together which metrics matter most and why. A short summary paragraph at the start or end of the Results would help.

Thank you for the suggestion, we have added a summary paragraph to the start of the Results (l.107-114). We hope the reviewer agrees that this enhances the readability and flow of the article.

The discussion section has a comprehensive coverage of replication successes, novel findings, null results, methodological limitations, and future directions. The acknowledgment of limitations is transparent and future recommendations for multimodal validation, standardized pipelines, are all appropriate. The biggest problem of the manuscript in my opinion is the fragmentation of the discussion section which reads like an extended results commentary rather than a conceptual synthesis.

Theoretical integration is underdeveloped: EBH is mentioned but not critically evaluated in light of findings, nor are alternative models explicitly linked to results. Null findings are acknowledged but not deeply interpreted—do they challenge EBH? The theoretical context could be expanded to include alternative models (e.g., REBUS, network-control theory).

Thank you for pointing out this clear omission from the Discussion, we have now added a paragraph directly reflecting on our implications for the EBH and suggestions for future work. (l.392-406). As discussed above, network-control theory in the section on meta-state complexity where we believe it is most relevant.

Reviewer #2 (Remarks to the Author):

The authors have addressed all of my comments.

Thank you for taking the time to review our manuscript.

Reviewer #4 (Remarks to the Author):

This paper provides a comprehensive comparison of the psychedelic effects on various measures of brain entropy derived from fMRI data. Psychedelic effects on brain activity have been a topic of growing interest, with increasing evidence suggesting potential clinical benefits in treating psychiatric disorders. Entropy is a useful metric for characterizing dynamic changes in brain states and their temporal evolution. This study recruited a new cohort to serve as an independent test sample for examining brain entropy under the influence of a psychedelic drug, which is an important strength. The authors further assessed the impact of fMRI preprocessing choices and compared 14 different entropy metrics—an analysis that has not been previously conducted. A strength is the use of longitudinal design. The comprehensive comparison is considered a strength but raises a concern of multiple comparison correction though many of metrics are quite different in nature. Below are the major and minor concerns.

Novelty is considered moderate though confirmation studies are also important but would ideally be based on more controlled data with a good sample size.

Although these metrics are all labeled as entropy measures, many differ substantially in their underlying definitions. For example, some capture temporal entropy, whereas others characterize entropy of spatial relationships between brain regions or the topological properties of connectomes. Some are related to Shannon entropy, while others reflect differential entropy. Therefore, obtaining different or inconsistent results across these metrics is not surprising.

We absolutely agree with the reviewer on this point. We believe our paper makes clear that these metrics, while each capturing entropy or complexity in some form, are separate constructs. This is valuable because it highlights that prominent models such as the Entropic Brain Hypothesis are problematically vague. We hope the reviewer agrees that this underscores the timeliness of our manuscript.

Data acquired from Scanner B has much higher spatial and temporal resolution and longer time series than those acquired from Scanner A. Entropy measures are highly sensitive to these parameters when applied to fMRI data. For example, the histogram-based Shannon entropy, LZ, and Sample En will have a systematic difference if the time series length is different. Higher temporal resolution will capture higher frequency signal. As entropy reflects irregularity, higher temporal resolution likely will provide more stable entropy estimates. This contrast can be seen from the rsfmri entropy studies based on the traditional non-multi-band bold fmri data and multi-band fmri data such as HCP. I would suggest the authors analyze these two subcohorts separately.

Thank you for the suggestion. Critically, we employ a within-subject design and each subject was scanned on only one scanner with the same scan parameters, including time-series length. Furthermore, “scanner” was included as a binary nuisance regressor in all statistical models in an effort to account for scanner effects on the relation between plasma drug levels and entropy.

Further, we kindly point the reviewer to our Results section “Moderating effect of scanner” and Supplementary Table S6, where we evaluate moderating effects of scanner on the association

between PPL and entropy metrics. Ideally all scans would have been acquired within a single scanner environment, yet we hope this provides a transparent assessment of associated scanner effects.

Otherwise, many entropy metrics are not combinable given the substantial differences of the data properties. If you want to do that, the only way will be downsampling cohort B to match the data from cohort A though you will still see systematic effects caused by the hemodynamic response function sampling difference, SNR and others.

We agree with the reviewer that alignment of sampling time is critical, that is why we did in fact already downsample the slower scans. Please see Methods section “Preprocessing” (l-521-522).

Coils are also very different in terms of SNR and regional inhomogeneity, which can be partially controlled through harmonization for those temporal brain entropy measures. Many subjects scanned in Scanner B had significantly shorter time series than the others. This will cause big variations to the entropy measures.

We thank the reviewer for raising this important point. We agree that scanner hardware-related differences (e.g., coil) as well as variation in fMRI time-series length can influence entropy-based measures. In our manuscript, we approached harmonisation through common preprocessing and denoising and we downsampled time-series from the fast scanner to match the TR of the slow scanner. Notwithstanding, we include scanner as a binary nuisance regressor, which provides a composite first-order approximation including hardware-associated variation, and evaluated the moderating effect of scanner on PPL associations. We observed that some temporal entropy measures were related to the scanner, e.g., long-time-scale sample entropy. Notably, the observed effect was in the same direction for both scanners. Also notable, we did not find a moderating effect of scanner for the strongest PPL associations, i.e., DCC and NGSC. We have also added a section to our Limitations highlighting the variation in numbers of volumes across scans (l.437-441).

There is a positive correlation between the drug intensity and drug level and motions. Likely, part of the observed fmri entropy effects was contributed by the drug-induced data variations. This is in addition to the potential vascular contributions commented by previous reviewers.

We agree with the reviewer that motion constitutes a meaningful challenge for psychedelic fMRI research. We have attempted to limit the effect of motion via regression of motion parameters, scrubbing of high-motion volumes, aCompCor, and including motion in our statistical model. Encouragingly, we also show little correlation between each of our whole-brain metrics and motion, except a moderate negative correlation with LZcs. We have included a discussion of this on (l.430-434).

Each entropy metric involves empirical parameters. Should the author describe how those parameters were chosen? For connectome binarization, how was the threshold was chosen? LZ depends on a threshold too.

The reviewer raises a relevant point that we have considered in preparing our manuscript. In this study, we focused on replicating previously reported metrics exactly. To do so, we implemented the precise parameters described in the corresponding papers. Many of the methods were not sufficiently well described in the original manuscript, motivating us to develop the CopBET toolbox that we introduce here and think greatly benefits transparency and reproducibility in the field going forward–this is affirmed by feedback and use since its first distribution. We engaged with authors of the original to ensure that our hyperparameter selection was identical to those previously applied. We have now included a line in the methods to clarify this position (l.527-530).

As psychedelic drugs impact the whole brain dramatically, the global signal will be affected. Then GSR is really problematic. GSR itself can be an index for assessing the drug effects.

We agree with the reviewer that it is important to understand how GSR affects our results. We have replicated our original pipeline with the addition of GSR. Please see Supplementary Tables 8 and 9 for an overview of effects with and without GSR. Where GSR has had a substantial effect on our results, we have included this in the results section for each metric.

For illustrations, could the authors show the statistical comparison results between pre- and post-drug entropy (at the peak drug level)?

Although we appreciate the potential added value of observing the difference from predrug to peak, this is something that we intentionally did not include in the paper, since we believe that having a regression-type analysis scheme is more informative than predrug-vs-peak effects. Focusing on only pre and peak scans would diminish our dataset by 60%, undermining the purpose of the repeated measures design. The time-to-peak is approximately 100 minutes, and datapoints are coloured according to time in each scatter plot (Figs. 2-4,6). Therefore, readers can compare purple points with the black points in each scatter plot to get an estimate of the peak effect.

If the difference between NGSC and Von Neumann entropy is the data dimension reduction, why not merge them and use one metric to avoid confusion?

We agree with the reviewer's implicit sentiment that those two measures are redundant. We believe that is well articulated through structuring our manuscript as a replication effort of previous effects. We highlight this point in the Discussion (l.262-266), which we hope guides the field toward, as the reviewer rightfully suggests, use one metric to avoid confusion.

One interesting though might be underpowered question that the authors can explore is to see the value of these metrics for predicting the psychedelic effects through an ablation study.

We agree with the reviewer that an ablation study could indeed inform on the relative importance of metrics in describing the psilocybin experience. In our manuscript we chose to describe the correlation between the metrics, which, in conjunction with the individual metrics' relation to plasma drug levels and methodological (dis-)similarity, achieves a similar purpose. We believe that keeping metrics separate with regards to their relation to plasma drug levels ensures that they are also interpreted separately. A well-performing prediction model is not our objective - instead we aim to describe the methodological foundation of metrics to get an intuition of how (and how well) they describe brain function (mechanistic insight), and relate this to their sensitivity to the psilocybin experience.

The last sentence in abstract is obvious based on the different definitions of these metrics.

We agree with the reviewer here, and this is part of the intention of this paper. Several of the previous articles evaluating these metrics, and those that cite them, describe these metrics as if they provide convergent evidence for a singular construct. By highlighting the differences between these metrics, including theoretical and empirical considerations, we believe a key take home of our manuscript is that it is imperative for the field to appreciate these differences in order to move forward effectively.

Variations of the time gap between drug and fMRI should be discussed.

Participants are in a drug-affected state that we must consider to ensure data are collected in a safe and ethical manner. Doing so naturally introduces between-subject variation in the acquisition times vis-a-vis drug administration. Regardless, this is not a problematic feature for our statistical modelling as we model the relation between each entropy metric and plasma drug levels or subjective drug intensity which are collected together with the scan. We do not model the time since administration since 1) we believe that plasma drug levels and subjective drug intensity are *more* informative than time since administration, and 2) modeling time is difficult, since longer time does not necessarily mean stronger drug experience since all participants also return to baseline after peak.

Longer outside-scanner time could in theory alter the strength of the subjective experience. However, it should not alter plasma drug levels. Such a time-outside-scanner effect is difficult to model with our data and would likely require a completely separate, perhaps within-subject, cohort. Furthermore, from our personal experiences gathering this dataset and others outside the MR-scanner environment, we do not believe such an effect to be present due to the potency of the drug and the dose - participants experience a strong psychedelic trip regardless of being inside or outside the scanner.

Result summary in Abstract is seemly inconsistent with Discussion. More metrics mentioned in Abstract that have significant positive effects while discussion (line 235).

Thank you for pointing this out, In our Abstract we articulate five consistently significant metrics, one inconsistent and eight consistently null. In the Discussion, we have adjusted the text to clarify that we consider Meta-state complexity consistent and LZct inconsistent, which aligns with what is written in the Abstract.

The timing for acquiring the several fMRI datasets should be provided.

We are somewhat in doubt about this reviewer comment. If relevant, we are reluctant to express each acquisition time relative to drug administration for each of the 121 datasets acquired; we shade data points in plots by acquisition time and we do not see the utility of articulating each acquisition time point. Alternatively, if relevant, we state in the Methods the time period during which project data were acquired, i.e., "Data presented here were collected between 2018 and 2021." (l.493).

If both the timing and the intervals are different across the subjects, they should be modeled in the linear mixed model.

As discussed above, we intentionally do not include time in our statistical model as the relation between plasma dug levels or subjective drug intensity is, in our opinion, more biologically relevant. Modeling a linear relation between time since drug administration and imaging metric implies an effect that perpetually increases over time; this is inconsistent with what we know about the pharmacodynamics and corresponding perceptual effects of psilocybin, insinuating that its inclusion can introduce model misspecification. Including scan times e.g., by binning the scans into predrug, peak, downslope etc would require arbitrary binning definitions and remove the important between-subject differences in drug metabolism and pharmacodynamics.

Legends for all plots should mention that all timepoints from all subjects were pooled.

Thank you for the suggestion, we have included the following text in the figure legend for each plot with a scatter plot "Each scatter plot includes repeated measures (n = 121 scans) for each subject (n = 28 subjects)."

It will be also important to show the curve for all subjects instead of showing the scatter points.

We appreciate this suggestion from the reviewer. The scatter plots show drug intensity (PPL, SDI, or Occ) on the x-axis and partial residuals on the y-axis - these partial residuals were computed by regressing out all regressors except for the drug intensity metric, including the random subject effect. As such, the inter-subject differences have already been accounted for and regressed out, thereby rendering subject-level curves redundant. Such lines would add additional complexity to the scatter plots without, in our opinion, clear benefit.

Not sure whether EEG or eye tracking or other biological data such as EKG, heart rate, blood pressures were measured. Maybe the authors can add some discussions about those as they may provide more objective information about the psychedelic drug effects.

We have included in the Discussion that simultaneous EEG in future studies (l.352-353) as well as using physiological tracking for noise removal should be considered (l.445). However, we believe that PPL, a direct measure of the psychoactive constituent immediately after each scan, is likely more directly informative of drug state than physiological measurements. Certainly such additional measurements could help to more fully characterise the physiological effects of psilocybin.

It may be interesting to check entropy of the non-denoised data (only with motion correction).

We agree that such an investigation could be interesting; however, we believe denoising is essential to fMRI analysis and therefore outside the scope of our paper. Further, we already include a wide range of denoising pipelines, each of which includes parameters that we believe are reasonable.

Fig. S1 B shows an obvious outlier. It would be interesting to see the results after excluding that subject.

We appreciate the suggestion from the reviewer. Designation of a data point as an “outlier” is complicated.The participant in question has an atypical SDI and PPL temporal profile vis-a-vis other participants, yet those profiles align within the participant (compare Fig. S1A and B). This exemplifies our previous comment about individual differences in drug metabolism and indirectly supports that PPL and SDI are internally consistent markers of the drug state. Considering these factors and our knowledge of that session, we do not see a clear indication that the participant data was acquired inaccurately. We are apprehensive about excluding datasets because they are dissimilar to the others as this can conversely and inadvertently under-estimate true between-subject variance in our signals.Therefore, we have decided to not exclude this subject.

Reviewer #4 (Remarks on code availability):

code sharing is a strength

Thank you

Reviewer #5 (Remarks to the Author):

I co-reviewed this manuscript with one of the reviewers who provided the listed reports. This is part of the Nature Communications initiative to facilitate training in peer review and to provide appropriate recognition for Early Career Researchers who co-review manuscripts.

Thank you for taking the time to review our manuscript.

We would like to thank the reviewers for their thoughtful comments, which have undoubtedly improved the manuscript. Please find below our point-by-point response to reviewer comments in red text and with indication to associated changes made to the manuscript. We would like to note that we have made additional modifications in response to the Author Checklist document provided by the Editorial Team.

Reviewer Comments

Responses are written in Red

I appreciate the comprehensive response. While I considered novelty moderate, the paper is important regarding the continuous entropy and PPL monitoring and the results would confirm the link between imaging-derived metrics and neuronal (actually should be quasi-neuronal since vascular effects have not been delineated). I have some minor comments for the authors to consider: I thought including the many different entropy metrics with totally different meanings only confuse the readership. If the authors prefer this way, I would describe them more carefully. Importantly, some of them are entropy of brain activity, the others are secondary and irrelevant to brain activity or at least not directly related. Some of them rather reflect the anatomical or topological construct.

We thank the reviewer for the time taken to review our manuscript. We agree whole-heartedly with the reviewer that the range of metrics that have previously been reported as “brain entropy” following psychedelic is diverse and non-singular. We hope highlighting this important consideration is one of the key takeaways of our study.

Neurovascular contribution might need a bit more discussion. As shown in Del Mauro et al: https://doi.org/10.1002/jmri.28948Digital, resting state fmri derived brain entropy correlates with physiological indices. By controlling those non-neuronal effects, entropy is still related to cognitive function as previously reported in https://doi.org/10.1016/j.neuroimage.2021.117893. Similarly, the authors might check whether further including those indices in the analysis will affect the effects or not.

Thank you for bringing to our attention the previously reported relation between Sample Entropy and physiological confounders. Unfortunately, we do not have measures of blood pressure or respiration rate in this cohort. We address this potential confounder in the Discussion around Sample Entropy:

“Beyond denoising, regional SampEn has also been shown to be positively associated with systolic blood pressure in HCP data across subjects [49]. Although a between-subject association does not necessarily translate to within-subject drug effects, psilocybin acutely raises blood pressure [50] so residual blood-pressure-related variance is a plausible contributor to the SampEn changes we observed. Taken together, increases in sample entropy at short temporal resolutions may be a candidate biomarker for psychedelic effects, if they cannot be explained by, e.g., wakefulness state or physiological confounds.”

Motion handling is considered standard and effective. Meanwhile, motion could also be related to the neuronal drug effects.

We agree, and have included this in the limitations section of the Discussion:

"PPL and SDI were associated with increased motion in the scanner, see Supplementary Figure S4; although we included an estimate of motion as a covariate in our models, employed scrubbing, motion correction and denoising strategies, and show that motion was not positively associated with any whole-brain entropy metrics, we cannot rule out that motion confounds our reported effects. It has been reported in many groups that head motion is increased following psychedelic drug administration so this is not a limitation unique to our data [53]."

Preprocessing in Method section didn't say downsampling. Did I miss something there?

We kindly refer the reviewer to text in the Pre-processing section of the Methods:

"Two MR-scanners were used to acquire the data, and some functional data were temporally downsampled so that the sampling frequency was consistent across scan sessions"

and in the Supplementary Methods:

"Following preprocessing and denoising and to align the temporal frequency between the two scanners, scanner B time-series were temporally downsampled to match the TR of scanner A (i.e., 2s) using the MATLAB command 256 "downsample"

fMRI based brain complexity or entropy analysis has gained increasing interest and several of the early papers should be cited:
Yang et al. Neurobiol Aging 2012, 10.1016/j.neurobiolaging.2012.05.004
Wang et al. Plos one 2014, https://doi.org/10.1371/journal.pone.0089948
Smith et al, Brain imaging behav 2014, 10.1007/s11682-013-9276-6

Thank you for bringing these to our attention, they have been added to the Methods section on Sample Entropy.
