# Supplementary material for: Multi-metric evaluations of acute psychedelic effects on fMRI brain entropy
Source: Nat Commun. 2026 Jun 24;17:7940. doi: 10.1038/s41467-026-74215-5 (PMC13448839; doi:10.1038/s41467-026-74215-5)
Supplement: Supplementary file 3 [file 41467_2026_74215_MOESM2_ESM.pdf]

Description of Additional Supplementary Files

File name: Supplementary Data S1

Description: Summary of previous findings. Overview of previous studies evaluating psychedelic effects on fMRI brain entropy metrics. For each metric, we report the original publication, sample size, drug administration route, brain parcellation used, whether findings were regional or whole-brain, and the reported effect direction. Abbreviations: IV, intravenous; ROIs, regions of interest.

File name: Supplementary Data S2

Description: Regional entropy metric results using the generic denoising pipeline. Linear mixed-effects model results for regional entropy metrics (out-network connectivity distribution entropy, intra-network synchrony distribution entropy, dynamic conditional correlation entropy, and multi-scale sample entropy) using the original parcellation and generic denoising pipeline. For each region, we report estimates, 95% confidence intervals, t-statistics, uncorrected permutation p-values, and family-wise error rate corrected p-values (p_{FWER}) for plasma psilocin level (PPL), estimated serotonin 2A receptor occupancy (Occ_{2A}), and subjective drug intensity (SDI). Significance indicators: *** $p < 0.0001$, ** $p < 0.001$, * $p < 0.05$, NS not significant.

File name: Supplementary Data S3

Description: Whole-brain entropy metric results using the generic denoising pipeline. Linear mixed-effects model results for whole-brain entropy metrics (BOLD complexity, degree distribution entropy, geodesic distribution entropy, Von Neumann entropy, normalised global spatial complexity, motif-connectivity distribution entropy, LEiDA-state Markov-rate entropy, meta-state complexity, and integration/segregation-state distribution entropy) using the original parcellation and generic denoising pipeline. For each metric, we report estimates, 95% confidence intervals, t-statistics, and permutation p-values for PPL, Occ_{2A}, and SDI.

File name: Supplementary Data S4

Description: Correlations between whole-brain entropy metrics. Pairwise Pearson correlation coefficients and associated p-values between whole-brain entropy quantifications across all included scans. The 'Correlations' sheet contains correlation coefficients and the 'P-values' sheet contains corresponding uncorrected p-values. Abbreviations: LZct, temporal Lempel-Ziv complexity; LZcs, spatial Lempel-Ziv complexity; I/S state, integration/segregation state; NGSC, normalised global spatial complexity; LEiDA, leading eigenvector dynamics analysis.

File name: Supplementary Data S5

Description: Entropy metric results using a common atlas. Linear mixed-effects model results for all entropy metrics using a common parcellation (Schaefer-100 cortical atlas with Tian-16 subcortical regions) and the generic denoising pipeline. Regional and whole-brain metrics are presented in separate sheets. Statistics reported as in Supplementary Tables S2 and S3.

File name: Supplementary Data S6

Description: Scanner interaction effects. Results from linear mixed-effects models evaluating the moderating effect of scanner on the relationship between plasma psilocin level and entropy metrics. For each metric, we report separate effect estimates for Scanner A and Scanner B, the interaction effect p-value (uncorrected), and the main effect p-value (p_{FWER} for regional metrics, p_{perm} for whole-brain metrics) from the primary analysis.

File name: Supplementary Data S7

Description: Pre-processing parameters from previous studies. Comparison of fMRI pre-processing pipelines used in previous studies evaluating psychedelic effects on brain entropy metrics. For each metric, we report the original dataset, anatomical regressors, number of motion regressors, temporal filtering parameters, spatial smoothing kernel, and scrubbing parameters employed. Abbreviations: WM, white matter; CSF, cerebrospinal fluid; FWHM, full width at half maximum; FD, framewise displacement.

File name: Supplementary Data S8

Description: Summary of multiverse analysis. Qualitative summary of psilocybin effects on brain entropy metrics across the original atlases, common atlas, and six alternative denoising pipelines (global signal regression, no low-pass filter, 24 motion parameters, no scrubbing, strict scrubbing, and narrow bandpass filter). For each metric, we report the original findings and our findings across all analysis variations. Abbreviations: IV Psi, intravenous psilocybin; Oral Aya, oral ayahuasca; GSR, global signal regression; PsiFx, psilocybin effects (PPL, Occ_{2A}, and SDI collectively).

File name: Supplementary Data S9

Description: Full multiverse analysis results. Complete statistical results for all entropy metrics across six alternative denoising pipelines. Separate sheets present whole-brain and regional results for each pipeline variation: global signal regression, no low-pass filter, 24 motion parameters, no scrubbing, strict scrubbing, and narrow bandpass filter. For each metric, we report permutation p-values, Pearson's rho effect sizes, parameter estimates, 95% confidence intervals, and t-statistics for PPL, Occ_{2A}, and SDI.
