# Supplementary material for: Multi-metric evaluations of acute psychedelic effects on fMRI brain entropy
Source: Nat Commun. 2026 Jun 24;17:7940. doi: 10.1038/s41467-026-74215-5 (PMC13448839; doi:10.1038/s41467-026-74215-5)
Supplement: Supplementary file 4 [file 41467_2026_74215_MOESM1_ESM.docx]

Supplementary Material

**Supplementary Text**

1. Study Design
2. Plasma Psilocin Level Quantification
3. BOLD scanning parameters
4. BOLD data preprocessing
5. fMRI Quality Control and missing data
6. Partial Residual Plots
7. In-depth Comparison with Tagliazucchi 2015
8. Pre-registration
9. Funding
10. Copenhagen Brain Entropy Toolbox

**Supplementary Figures**

1. Time courses of subjective drug intensity and plasma psilocin level
2. Intra-network Synchrony Distribution regression plots
3. Tagliazucchi peak vs baseline plot replication
4. Relation between motion and PPL / SDI

**Supplementary Tables**

1. Previous papers
2. Regional metrics (max T corrected)
3. Whole-brain metrics
4. Correlation between whole-brain entropy metrics
5. Common atlas results
6. Moderating effect of scanner
7. Preprocessing pipeline comparisons
8. Summary of results across all preprocessing pipelines
9. Detailed results across all preprocessing pipelines

# Supplementary Text

## Study Design

All participants were screened to exclude individuals with significant somatic disease, neurological disorders and/or psychiatric disease using the Mini-International Neuropsychiatric Interview (Danish translation version 6.0.0). Prior to inclusion, participants received written and verbal descriptions of the study protocol, including the expected effects of psilocybin and potential side effects of administration; all participants provided written consent prior to study participation.

Exclusion criteria were 1) present or previous personal or family (first degree relatives) history of psychiatric disease (DSM axis 1 or WHO ICD-10 diagnostic classification) including substance misuse disorder; 2) Present or previous neurological condition/disease, significant somatic disease or any intake of drugs suspected to influence test results; 3) nonfluent Danish language skills; 4) vision or hearing impairment; 5) learning disability; 6) pregnancy; 7) breastfeeding; 8) MRI contraindications; 9) alcohol or drug abuse 10) allergy to test drugs; 11) significant exposure to radiation within the past year (e.g., medical imaging investigations); 12) intake of QT-prolonging medication or electrocardiogram (ECG) results indicative of heart disease, 13) history of significant adverse response to a hallucinogenic drug. 14) use of hallucinogenic drugs less than 6 months prior to inclusion; 15) blood donation less than 3 months before project participation; 16) body weight less than 50 kg; 17) low plasma ferritin levels (< 12 μg/L).

The order of psilocybin vs. ketanserin administration was balanced across participants as well as possible, drug sessions were separated by at least three weeks. The data presented here is part of a study approved by the ethics committee for the Capital Region of Copenhagen (journal identifier: H-16028698, amendments: 56023, 56967, 57974, 59673, 60437, 62255) and Danish Medicines Agency (EudraCT identifier: 2016-004000-61, amendments: 2017014166, 2017082837, 2018023295).

Participants were asked to refrain from alcohol the night before intervention days and to eat a light breakfast and refrain from caffeine intake on the morning of intervention. Prior to intervention days, a urine sample was drawn and screened for amphetamines, opioids, benzodiazepines, barbiturates, tetrahydrocannabinol, cocaine, ketamine, phencyclidine, and gamma hydroxybutyrate (Rapid Response, BTNX Inc., Markham, Canada), a blood sample and an electrocardiogram were taken to ensure that individuals were in good overall physiological health (e.g., absent evidence for a prolonged QT interval).

On the day upon which participants received psilocybin, they arrived in the morning, completed state questionnaires and a pre-drug MRI scan session. Following a brief conversation with the psychological support personnel (two people) present for the psilocybin administration day, participants received psilocybin in a room adjacent to the MRI scanner. Participants could lay on a bed or sit in a chair in this room and music was played after psilocybin administration but not during scanning.

Participants were financially compensated for the hours spent taking part in the study.

## Components of preparation, support and integration sessions

### Preparation:

1. The purpose of the session
2. The participant’s motivation for entering the study and expectancy management
3. The participant’s personal background
4. Previous experience with psychedelics or altered states of consciousness
5. Information about possible effects of psilocybin
6. Tools for navigating the psychedelic experience, with a particular focus on the MR environment
7. The participant's social support-network and potential contact person
8. Practical road map of the intervention day, including rules for the study
9. Agreements regarding therapeutic touch and physical support

### Intervention (before administration):

1. The purpose of the session, including any questions arising since preparation
2. Anchoring - and repetition of tools for navigating the experience
3. Support the participant in letting go of everyday tasks and preoccupations, including hand over of their mobile phone and helping with arrangements of various matters with the participant’s family/contact person or social network
4. Repeating the practicalities of the intervention, including rules and agreements made during preparation
5. Administration of psilocybin

### Intervention (end of day):

1. Drawing a mandala
2. Information about afterglow effects and making a plan for self-care upon arrival at home
3. Confirmation of time for integration session

### Integration:

1. The purpose of the session
2. Integration wheel
3. Eliciting a full narrative about psychedelic experience
4. Witnessing and debriefing of difficult material
5. Relating the psychedelic experience to the participant’s life going forward
6. Plan for self-care
7. Thoughts about ending the study and information about the possibility of contacting the psychologists, if needed
8. Any questions that the participant may have

## Plasma Psilocin Level Quantification

Plasma psilocin level was measured using ultra performance liquid chromatography and tandem mass spectrometry, reflecting free, unconjugated psilocin and not glucuronidated psilocin ^2^.

7 mL blood samples were drawn from an intravenous access in the antecubital vein and collected in EDTA vials, placed on ice, centrifuged, and plasma was aliquoted and stored at -20 or -80°C. Psilocin was obtained from Lipomed (Arlesheim, Switzerland) and Cerilliant (Round Rock, TX, USA) for calibrator and control batches, respectively, while the deuterated internal standard (IS) psilocin-d10 was from Cerilliant. Acetonitrile, methanol HPLC grade and water were obtained from Fishers Scientific (Loughborough, UK). Ascorbic acid was obtained from VWR (Hassrode, Belgium).

Stock solution (1000 mg/l) of psilocin and IS were prepared in acetonitrile and stored in amber ampoules at -20°C until use. Working standard solutions from 0.5 µg/l to 1000 µg/l were freshly prepared in 50% methanol in water for each analysis, and the IS-solution was 100 µg/l in 50% methanol. For preparation of calibration standards and quality controls blank plasma was preserved with 1% fluoride and stored at -20°C. Two quality controls (QC) were prepared at low (5.0 µg/kg) and high (50 µg/kg) levels and stored at -80°C. These two along with a freshly spiked blank plasma sample at 2.5 µg/kg were analysed in each run.

Protein precipitation was performed on a fully automated Tecan Freedom EVO 200 robotic platform (Tecan group Ltd, Männedorf, Switzerland) that included all pipetting, centrifugation, and evaporation steps. Each plasma sample (100 mg) was transferred to a 96-well 2.0 ml deep-well plate and 20 µl IS-solution was added to each well, followed by precipitation with 700 µl acetonitrile and shaking. The samples were centrifuged at 1000 g for 10 min, and the supernatant was evaporated to dryness under a stream of nitrogen at 35°C. Afterwards, the samples were reconstituted in 100 µl mixture of 12.5% methanol:12.5% acetonitrile:75% 0.05% formic acid in water, shaken and centrifuged again. Finally, the supernatant was transferred to a 96-well plate and 1 µl was injected into the chromatographic system.

Chromatographic separation was performed on a HSS T3 column (100 x 2.1 mm, 1.8um, Waters, Milford, MA, USA) using an ACQUITY Ultra Performance Liquid Chromatography system (UPLC) from Waters. The mobile phase was composed of solvent A: 1 mM ammonium formate in 0.1% formic acid in water and B: 0.1% formic acid in 1:1 mixture of acetonitrile:methanol. The column was maintained at 45°C with a flow 0.4 ml/min, and a gradient elution was applied from 2% to 100% B within 3.2 min with a total analysis time of 4.5 min. Detection was done by tandem mass spectrometry using an ACQUITY TQS from Waters. Ionisation was achieved by electrospray in positive mode, and the source temperature was set at 150°C and desolvation temperature at 600°C. Two transitions were used for psilocin, m/z 205 -> 58 and 205 -> 160, with a cone voltage of 20 V and collision energy at 14 and 18 eV, respectively. For the IS the transition was m/z 215 -> 164 with cone 20 V and collision energy of 18 eV. Argon was used as collision gas at 0.45 Pa, and desolvation and cone gasflow were fixed at 1000 L/hr and 150 L/hr, respectively. Data were acquired and processed with MassLynx 4.2 software (Waters).

Quantification was performed by an eight-point linear calibration curve (0.1, 0.5, 1.0, 5.0, 25, 50, 100, 200 µg/kg) with weighting 1/x. Limits of detection (LOD) and quantification (LOQ) were 0.1 and 0.5 µg/kg, respectively, while the upper limit of quantification was 200 µg/kg. The overall process efficiency was found to be 63% based on an obtained extraction efficiency of 81% and matrix effect of 25% that the stable isotope labelled IS adjusted for. QC plasma samples were measured in each series with a RSD of 5% and an accuracy of 78% for the low level and less than 5% and an accuracy 89% for the high level. Similar performance was obtained with QCs preserved with 1 mM ascorbic acid demonstrating that stored plasma QCs at -80°C without ascorbic acid were stable for at least 6 months. The freshly spiked plasma QC at 2.5 µg/kg analysed in each series had a RSD of 13% and an accuracy of 88% (n = 11). For the final presentation of results, units of concentration (µg/kg) were converted into µg/L using a conversion factor or 1.02 kg per litre of plasma.

## BOLD Scanning Parameters

Due to logistical constraints, MRI scans were acquired on one of two Siemens 3T MAGNETOM Prisma scanners (“Scanner A”, “Scanner B”). The first 15 participants were scanned on Scanner A, the final 13 participants were scanned on Scanner B, all participants completed all scans on a single scanner.

A high-resolution, T1-weighted 3D MPRAGE structural image was acquired (Scanner A: inversion time = 900 ms, TE = 2.58 ms, TR = 1900 ms, flip angle = 9°, in-plane matrix = 256x256, resolution = 0.9x0.9 mm, 224 slices; slice thickness = 0.9 mm, no gap, Scanner B: inversion time = 920 ms, TE = 2.41 ms, TR = 1810 ms, flip angle = 9°, in-plane matrix = 288 × 288, in-plane resolution = 0.8×0.8 mm, number of slices = 224, slice thickness = 0.8 mm).

BOLD fMRI data was obtained using a T2*-weighted gradient echo-planar imaging (EPI) sequence.

Scanner A: 64-channel head coil, TR = 2000 ms, TE = 30 ms, flip angle = 90°, in-plane matrix = 64x64 voxels, in-plane resolution=3.6x3.6 mm, number of slices = 32, slice thickness = 3.0 mm, gap = 0.75 mm, phase-encoding direction = AP, scan time = 10 minutes, volumes = 300.

Scanner B: 32-channel head coil, TR = 800 ms, TE = 37 ms, flip angle = 52°, in-plane matrix = 104x104 voxels, in-plane resolution = 2x2 mm, slices = 72, slice thickness = 2.0 mm, gap = 0, multi-band acceleration factor = 8, scan time = 10 minutes, volumes = 750 (n = 5) or 375 (n = 8, due to an acquisition error).

## BOLD Data Preprocessing

BOLD data were preprocessed in SPM12 (http://www.fil.ion.ucl.ac.uk/spm) and denoised using the CONN toolbox ^3^ using MATLAB (The Mathworks, inc., version 2019b 9.7.0). Preprocessing pipelines were identical for both scanners unless specified. We applied slice-timing correction (scanner A only because scanner B used multi-band), data were unwarped and realigned, structural scans were co-registered to functional data. High-resolution structural images were segmented into grey-matter, white-matter and CSF-maps; functional data were normalised to MNI152 space based on parameters estimated during the segmentation procedure and smoothed using a 6mm FWHM gaussian kernel. Denoising included linear detrending, aCompCor regression of white-matter and CSF time-series (first five components for each and their first derivatives) ^4^ as well as regression of 6-motion parameters and their first derivatives, and scrubbing of volumes flagged using the artifact detection tool (ART, <https://www.nitrc.org/projects/artifact_detect>, global signal > 4 or motion > 2). Some analyses utilised unsmoothed data to align with previous methods (Intra-network synchrony, Motif-connectivity distribution, Multi-scale Sample Entropy.). A bandpass filter of 0.008 - 0.09 Hz was applied. Regional time-series were averaged in CONN using analysis-specific atlases. Following preprocessing and denoising and to align the temporal frequency between the two scanners, scanner B time-series were temporally downsampled to match the TR of scanner A (i.e., 2s) using the MATLAB command “downsample”.

## fMRI Quality Control and Missing Data

All BOLD data were visually inspected to ensure accurate co-registration to MNI space. Connectivity matrices and regional time-series were visually inspected for artefacts or missing data.

Of the 130 resting-state fMRI scan sessions acquired during psilocybin intervention days for the 28 participants (76 Scanner A and 54 Scanner B), Nine scans and no participants were excluded from analyses: two scans from one participant because of excessive head motion through the scan session (>50% of volumes flagged for censoring), one scan from one participant due to technical problems with data acquisition, two scans from one participant due to 1) interrupted scan and 2) technical problems with data acquisition, one scan each from three participants due to interrupted scans. Thus, 121 scan sessions were included in analyses. One scan session did not have an accompanying measure of PPL, another was without a measure of SDI, these individual scans were excluded from these respective analyses.

## Partial Residual Plots

To display the relation between PPL and each whole-brain entropy metric, plots were constructed that display PPL values (in μg/L) on the x-axis and adjusted residual entropy values on the y-axis. These are adjusted by removing the covariate effects. This was performed by taking the raw entropy score and deducting the beta of each covariate multiplied by the relevant value of that covariate for each scan.

## In-depth Comparison with Tagliazucchi 2015

We do not observe a significant relation between psilocybin effects and hippocampal-ACC motif distribution entropy, calculated as described in ^5^. The original publication describes a significant difference comparing both pre-psilocybin vs post-psilocybin and post-psilocybin vs post-placebo at longer (c.75-150 second) windows. Unfortunately, the entropy values presented by Tagliazucchi and colleagues leave significant cause for concern. They use non-overlapping windows, the shortest window-lengths they used were 10-seconds, and their scans were 300-seconds long. Thus, at the shortest window lengths they would observe 30 brain states whereas at the longest window length (150-seconds) only two events would be observed. Thus at the longest window-lengths, there are two possible Shannon entropy values, one (i.e., two different states -2 x 0.5 x log2(0.5) = 1) and zero (i.e., two of the same state -1 x 1 x log2(1) = 0). Thus, it is mathematically impossible to have Shannon entropy values in excess of 1, which are reported in this original publication. Additionally, one would expect the Shannon entropy values at short window-lengths to be higher than those at long window-lengths, as there are more events in the histogram of motifs (i.e., 5 of state 1, 3 of state 2, 10 of state 3, 6 of state 4, 6 of state 5, Shannon entropy = 2.2 bits) but they report window-lengths approaching zero as window length approaches 10-seconds. Although unlike the high entropy values at long window length, this is mathematically possible, as the same state may reoccur with increasing frequency at shorter window-lengths (i.e., 29 of state 1, 1 of state 2, Shannon entropy = 0.21), but this seems highly unlikely and is contrary to what we observe (Supplementary Figure S3).

## Pre-registration

This study was pre-registered on the 3rd of August 2022 and the pre-registration document can be viewed here https://aspredicted.org/bw8y7.pdf. Notably, three of 13 papers were identified after pre-registration and therefore not described therein.

## Funding

The collection of the data reported here, but not the current analysis work was supported by Innovation Fund Denmark (grant number 4108–00004B), Independent Research Fund Denmark (grant number 6110–00518B), and Ester M. og Konrad Kristian Sigurdssons Dyreværnsfond (grant number 850–22–55,166–17-LNG). MKM was supported during data collection through a stipend from Rigshospitalet’s Research Council (grant number R130-A5324). BO has received funding from the European Union’s Horizon 2020 research and innovation program under the Marie Sklodowska-Curie grant agreement No 746,850. DEWM salary is supported by COMPASS Pathways Ltd. The funders had no role in study design, data collection and analysis, decision to publish or preparation of the manuscript.

## Inclusivity and Ethics statement

All aspects of this study were conducted with rigorous adherence to ethical guidelines as evaluated by the Ethics Committee for the Capital Region of Copenhagen and Danish Medicines Agency. Data management was undertaken with utmost care. The secure CIMBI database was employed for all data storage, ensuring participant confidentiality and robust protection of personal data. The conduct of the clinical trial was overseen by the Good Clinical Practice (GCP) unit, ensuring that all procedures adhered to the highest ethical and professional standards.

## Copenhagen Brain Entropy Toolbox

The twelve described entropy metrics have been collected in the MATLAB-based “Copenhagen Brain Entropy Toolbox” (CopBET) located here: <https://github.com/anders-s-olsen/CopBET>. The toolbox was developed in MATLAB R2018b and includes external dependencies to the Brain Connectivity Toolbox (BCT) v19.03.03 (https://sites.google.com/site/bctnet/), the Complexity Toolbox (LOFT) (https://loft-lab.org/index-5.html), and Martin Lindquist’s Dynamic Conditional Correlation toolbox (https://github.com/canlab/Lindquist_Dynamic_Correlation/tree/master/DCC_toolbox). The CopBET has further been tested to ensure functionality using Matlab versions R2019b and R2023a. However, it does not work using versions prior to R2018b. The CopBET contains individual functions for each of the 13 metrics as well as a script that may be used to test the functions using example data.

An example call to the toolbox could be:

>> output_table = CopBET_function(input_table)

Here, ‘input_table’ is a MATLAB-table where each row in the first column contains a regional timeseries, i.e., is a matrix n x p, where n is the number of acquired samples for the scan in question, and p is the number of regions in the atlas used for parcellation. Three of the functions take the path to (denoised) voxel-wise timeseries (4D-nifti file) as inputs instead of regional timeseries. The output, ‘output_table’ will consist of entropy-values for each scan in ‘input_table’. The function name ‘CopBET_function’ may be replaced with the name of any of the 12 included functions. All atlases described in this paper are also included in the repository in 2mm MNI152 space. Each function outputs either one or several entropy values for each scan, e.g., for each "mean degree” for the degree distribution entropy.

# Supplementary Figures

**Figure S1**: Time-course of (A) plasma psilocin levels and (B) subjective drug intensity per participant. Each colour represents a participant and the black line represents a locally estimated scatter-plot smoothing across the sample. Pre-drug PPL measurements are set at 0 mins. fMRI scans were performed before drug administration and then at approximately +40, +80, +120 and +300 minutes.

**Figure S2**: Scatter plots describing the relation between variance of intra-network synchrony (VOINS) of dynamic BOLD activity and plasma psilocin level, for each network: A) Dorsal attention, B) Default-mode, C) Left Frontoparietal, D) Right Frontoparietal, E) Salience, F) Visual1, G) Visual2, H) Auditory, I) Motor. Y-axis values are adjusted for age, sex, MR scanner and motion. All p_FWER_ > 0.98. Each scatter plot includes repeated measures (n = 121 scans) for each subject (n = 28 subjects). Statistical associations were tested using two-sided permutation-based linear mixed-effects regression. P-values are not corrected for multiple comparisons.

**Figure S3:** Scatter plot of motif-connectivity distribution entropy with overlaid locally estimated scatter-plot smoothing showing the relation between window-size and entropy derived from the linear model. Blue points and line represent the intercept value from the linear model comparing PPL and entropy (i.e., model estimated entropy when PPL = 0). The red points and line represent model estimated entropy when PPL = 15 μg/L, approximately PPL at peak drug effects. Note the discrepancy in y-axis values and that we observed decreasing entropy with increasing window size vis-a-vis Figure 5A from Tagliazucchi et al., 2015.

**Figure S4:** Scatter plots displaying the adjusted linear relation between motion (i.e., framewise displacement, FD) and (A) PPL or (B) SDI. Y-axis values are partial residuals adjusted for covariates, i.e., age, sex, scanner and subject. P-values are uncorrected. Pearson’s Rho are 0.33 for PPL and 0.43 for SDI.

## References

1.	Knudsen, G. M. *et al.* The Center for Integrated Molecular Brain Imaging (Cimbi) database. *NeuroImage* **124**, 1213–1219 (2016).

2.	Kolaczynska, K. E., Liechti, M. E. & Duthaler, U. Development and validation of an LC-MS/MS method for the bioanalysis of psilocybin’s main metabolites, psilocin and 4-hydroxyindole-3-acetic acid, in human plasma. *J. Chromatogr. B* **1164**, 122486 (2021).

3.	Whitfield-Gabrieli, S. & Nieto-Castanon, A. Conn: A Functional Connectivity Toolbox for Correlated and Anticorrelated Brain Networks. *Brain Connect.* (2012) doi:10.1089/brain.2012.0073.

4.	Behzadi, Y., Restom, K., Liau, J. & Liu, T. T. A component based noise correction method (CompCor) for BOLD and perfusion based fMRI. *NeuroImage* (2007) doi:10.1016/j.neuroimage.2007.04.042.

5.	Tagliazucchi, E., Carhart-Harris, R., Leech, R., Nutt, D. & Chialvo, D. R. Enhanced repertoire of brain dynamical states during the psychedelic experience. *Hum. Brain Mapp.* **35**, 5442–5456 (2014).
